# Supplementary material for: Evaluation of patients’ satisfaction with bronchoscopy procedure
Source: PLoS One. 2022 Oct 6;17(10):e0274377. doi: 10.1371/journal.pone.0274377 (PMC9536568; doi:10.1371/journal.pone.0274377)
Supplement: S1 Table — (PDF) [file pone.0274377.s001.pdf]

Questionnaire B (before bronchoscopy)

The first questionnaire was conducted 24 hours before the procedure. Its main aim was to assess patient anxiety on the day preceding bronchoscopy and patient satisfaction with the information provided by their attending physician.

No.	Question	Possible answers
Q1	Have you been informed about the reason for undergoing bronchoscopy tomorrow?	"yes"<br>"rather yes"<br>"partly"<br>"rather not"<br>"no"
Q2	Have you been informed about the course of the bronchoscopy?	"yes"<br>"rather yes"<br>"partly"<br>"rather not"<br>"no"
Q3	Have you been informed about the possible complications during bronchoscopy?	"yes"<br>"rather yes"<br>"partly"<br>"rather not"<br>"no"
Q4	Did you receive any written information about bronchoscopy?	"yes"<br>"no"
Q5	Was the overall information given to you sufficient for you?	"yes"<br>"rather yes"<br>"partly"<br>"rather not"<br>"no"
Q6	Do you or did you smoke, and if you do/did, how many cigarettes do/ did you smoke daily, for how many years?	"yes"<br>no"

Q7	On a scale of 1-10, what is your level of anxiety concerning your bronchoscopy?	0 1 2 3 4 5 6 7 8 9 10<br>↑ ↑<br>No anxiety or fear — Severe anxiety
Q8	Have you ever had a bronchoscopy before and if yes, how many times did you undergo this procedure and how would you rate your satisfaction during and after it on a scale of 1-10	0 1 2 3 4 5 6 7 8 9 10<br>↑ ↑<br>No bad experiences — Severe discomfort during or after
