# Supplementary material for: Evaluation of patients’ satisfaction with bronchoscopy procedure
Source: PLoS One. 2022 Oct 6;17(10):e0274377. doi: 10.1371/journal.pone.0274377 (PMC9536568; doi:10.1371/journal.pone.0274377)
Supplement: S2 Table — (PDF) [file pone.0274377.s002.pdf]

"Questionnaire P (post bronchoscopy)"

24-36 hours after their bronchoscopy, patients who had completed the first questionnaire were asked to complete another one. The aim of this survey was to assess patients' satisfaction with their procedure, learn about the factors that contributed to their anxiety or discomfort and finally ask whether the information they received from their attending physicians was adequate to their experience.

The questions asked in the second survey were as follows:

No.	Question	Possible answers
Q1	On a scale of 1-10, how would you rate your satisfaction after bronchoscopy?	0 1 2 3 4 5 6 7 8 9 10<br>↑ (0) Very satisfied<br>↑ (10) Very unsatisfied
Q2	Would you agree to a reexamination if necessary?	"yes"<br>"rather yes"<br>"partly"<br>"rather not"<br>"no"
Q3	Do you remember the course of your procedure?	"yes"<br>"rather yes"<br>"partly"<br>"rather not"<br>"no"
Q4	Select 3 factors that caused the biggest discomfort during your bronchoscopy	"local anesthesia"<br>"cough"<br>"retention and runoff of secretions/saliva"<br>"dyspnea"<br>"chest pain"<br>"pain located elsewhere" (please specify where)<br>"nausea"<br>"impaired consciousness"<br>"other" (please specify)

Q5	Select 3 factors that caused the biggest discomfort during your bronchoscopy	“I did not experience anxiety”<br>“lack of information before the procedure”<br>“information from other patients”<br>“appearance of the room and the equipment”<br>“preparation before the procedure (fasting, inserting an IV cannula)”<br>“lack of support from medical staff (nurses and doctors)<br>“premedication”<br>“local anesthesia of the throat”<br>“discomfort on inserting the bronchoscope”<br>“complaints during the procedure itself”<br>“complaints after the procedure, please specify”<br>“others, please specify”
Q6	Was your anxiety before the procedure adequate to your experience?	“No, the course of the procedure was harder than I expected.”<br>“No, the course of the procedure was better than I expected”<br>“Yes, the procedure met my expectations”
Q7	Did the information given to you by the medical staff correspond with what you experienced?	“No, the information was not sufficient”<br>“No, the information was too detailed and caused excessive anxiety”<br>“Yes, the information was appropriate”
