# Supplementary material for: Evaluation of patients’ satisfaction with bronchoscopy procedure
Source: PLoS One. 2022 Oct 6;17(10):e0274377. doi: 10.1371/journal.pone.0274377 (PMC9536568; doi:10.1371/journal.pone.0274377)
Supplement: S3 Table — (PDF) [file pone.0274377.s003.pdf]

***Course of bronchoscopy***

The third questionnaire was completed by bronchoscopists and was aimed at gathering crucial information about the course and duration of the procedure and potential complications.

The questions asked in this survey were as follows:

| No. | Question | Possible answers |
|---|---|---|
| Q1 | Type of bronchoscopy | "videobronchofiberscopy",<br>"videobronchofiberoscopy +EBUS (radial)",<br>"videobronchofiberoscopy + EBUS (sectoral)",<br>"rigid bronchoscopy", "bronchoscopy through tracheostomy" |
| Q2 | Indication for bronchoscopy | "suspected lung tumor or lung nodule"<br>"interstitial lung abnormalities"<br>"recurrent pulmonary infections"<br>"pneumonia/bronchitis not responding to treatment"<br>"suspected tuberculosis or mycobacteriosis"<br>"bronchiectasis"<br>"hemoptysis"<br>"dyspnoea of unclear etiology"<br>"cough of unclear etiology"<br>"suspicion of an inhaled foreign body"<br>"assessment of bronchial patency before planned surgery"<br>"endobronchial toilet"<br>"mediastinal lymphadenopathy"<br>"others, please specify" |
| Q3 | Premedication | "no premedication"<br>"midazolam p.o."<br>"atropine i.m."<br>"others" (please specify)<br>premedication execution time |
| Q4 | Type of anesthesia | "lidocaine"<br>"midazolam"<br>"fentanyl"<br>"general anesthesia" |

| | | |
|---|---|---|
| Q5 | Duration of application of local anesthesia (if applicable) and duration of bronchoscopy (min). | |
| Q6 | Oxygen administration | “no”<br>“yes”, oxygen flow (l/min ) |
| Q7 | Oxygen saturation and heart rate | $SpO_2$ and HR at the beginning and at the end of the procedure.<br>Duration of $SpO_2$ drop < 90% and duration of $SpO_2$ drop of > 4%. |
| Q8 | Procedures during bronchoscopy | “bronchial washing”<br>“bronchoalveolar lavage (BAL)”<br>“bronchial forceps biopsy”<br>“bronchial brush biopsy”<br>“transbronchial lung/tumor biopsy”<br>“endobronchial ultrasound-guided transbronchial needle aspiration (EBUS-TBNA)”<br>“radial endobronchial ultrasound-guided transbronchial biopsy (rEBUS-TBB)”<br>“tracheobronchial toilet”<br>“foreign body removal” |
| Q9 | Complications | “no complications”<br>“bleeding- mild; moderate; severe”<br>“arrhythmia during bronchoscopy (AF, SVT/VT, extrasystoles)”<br>“tachycardia >120/min”<br>“choking”<br>“drop of $SpO_2$ <85%”<br>“tachypnoe >30/min”<br>“bradypnoe < 8/min”<br>“others, please specify” |
| Q10 | Problems during the course of bronchoscopy | “no problems”<br>“lack of compliance”<br>“agitation”<br>“severe cough”<br>“excessive salivation and secretion from the airways”, |

| | | |
|---|---|---|
| | | “severe bleeding”<br>“others” (please specify) |
