# Supplementary material for: Evaluation of patients’ satisfaction with bronchoscopy procedure
Source: PLoS One. 2022 Oct 6;17(10):e0274377. doi: 10.1371/journal.pone.0274377 (PMC9536568; doi:10.1371/journal.pone.0274377)
Supplement: S4 Table — (PDF) [file pone.0274377.s004.pdf]

“Questionnaire for physicians after bronchoscopy"

The last questionnaire was completed by the patients' attending physician. The patient had undergone bronchoscopy 48 hours before. The aim of this survey was to learn about the complications in the first 48 hours following the procedure and the medical history of the patient.

The questions asked in the last survey were as follows:

No.	Question	Possible answers
Q1	Were there any complications in the first 48 hours after bronchoscopy?	“hemoptysis persisting 4 hours after the procedure”<br>“sore throat requiring pain management”<br>“fever persisting 12 hours after the procedure”<br>“pneumonia”<br>“asthma/COPD exacerbation”<br>“respiratory failure or exacerbation of previous respiratory failure”<br>“pneumothorax”<br>“chest pain requiring pain management”<br>“pain located elsewhere, please specify”<br>“impaired consciousness”<br>“breathing problems”<br>“arrhythmia, please specify”<br>“acute coronary syndrome”<br>”hoarseness or dysphonia persisting 12 hours after bronchoscopy”<br>“others, please specify”
Q2	Has the patient ever been treated for any of those diseases?	“chronic obstructive pulmonary disease (COPD)”<br>“asthma”<br>“idiopathic pulmonary fibrosis”<br>“tuberculosis”<br>“lung tumor”<br>“other tumors, please specify”<br>“chronic heart failure”<br>“ischemic heart disease (coronary)”<br>“hypertension”<br>“arrhythmia”<br>“diabetes”<br>“peptic ulcer disease or gastroesophageal reflux disease”<br>“chronic sinusitis”

		“hematological diseases, please specify”<br>“neurological diseases, please specify”<br>“others, please specify”
