# Supplementary figures and images for: Evaluation of patients’ satisfaction with bronchoscopy procedure
Source: PLoS One. 2022 Oct 6;17(10):e0274377. doi: 10.1371/journal.pone.0274377 (PMC9536568; doi:10.1371/journal.pone.0274377)

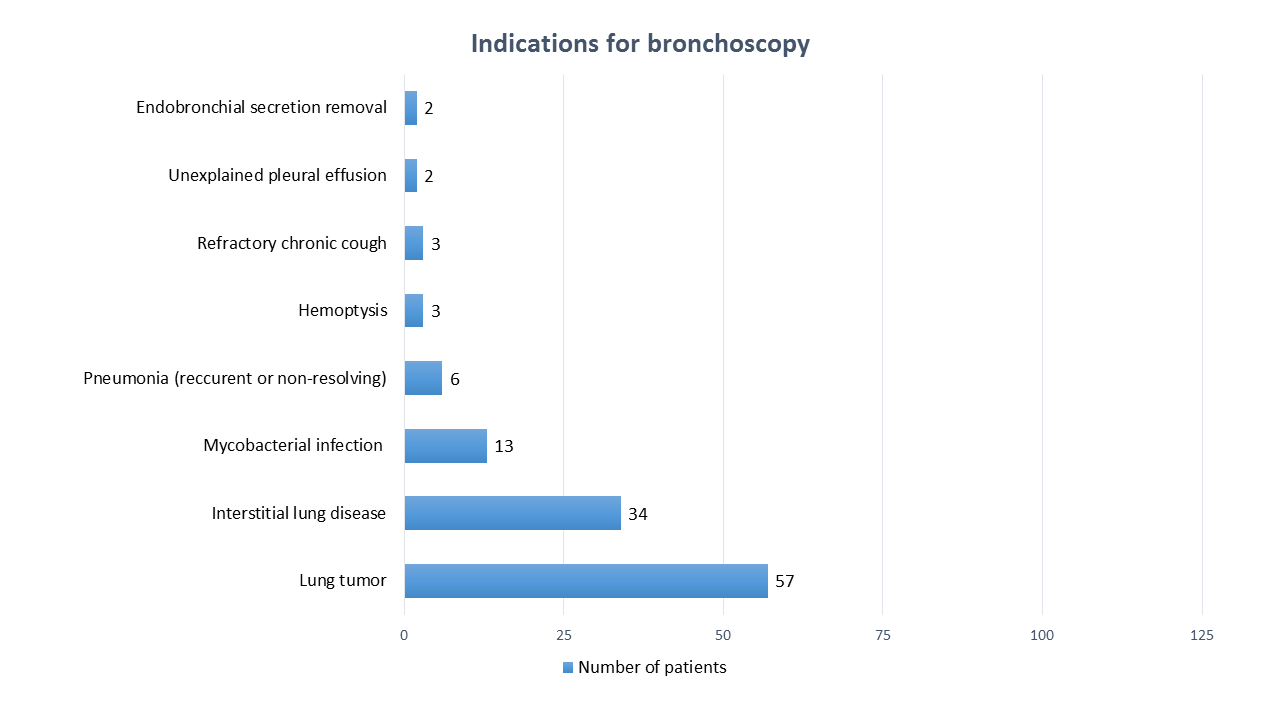

Supplement: S1 Fig — (TIF) [file pone.0274377.s005.tif]

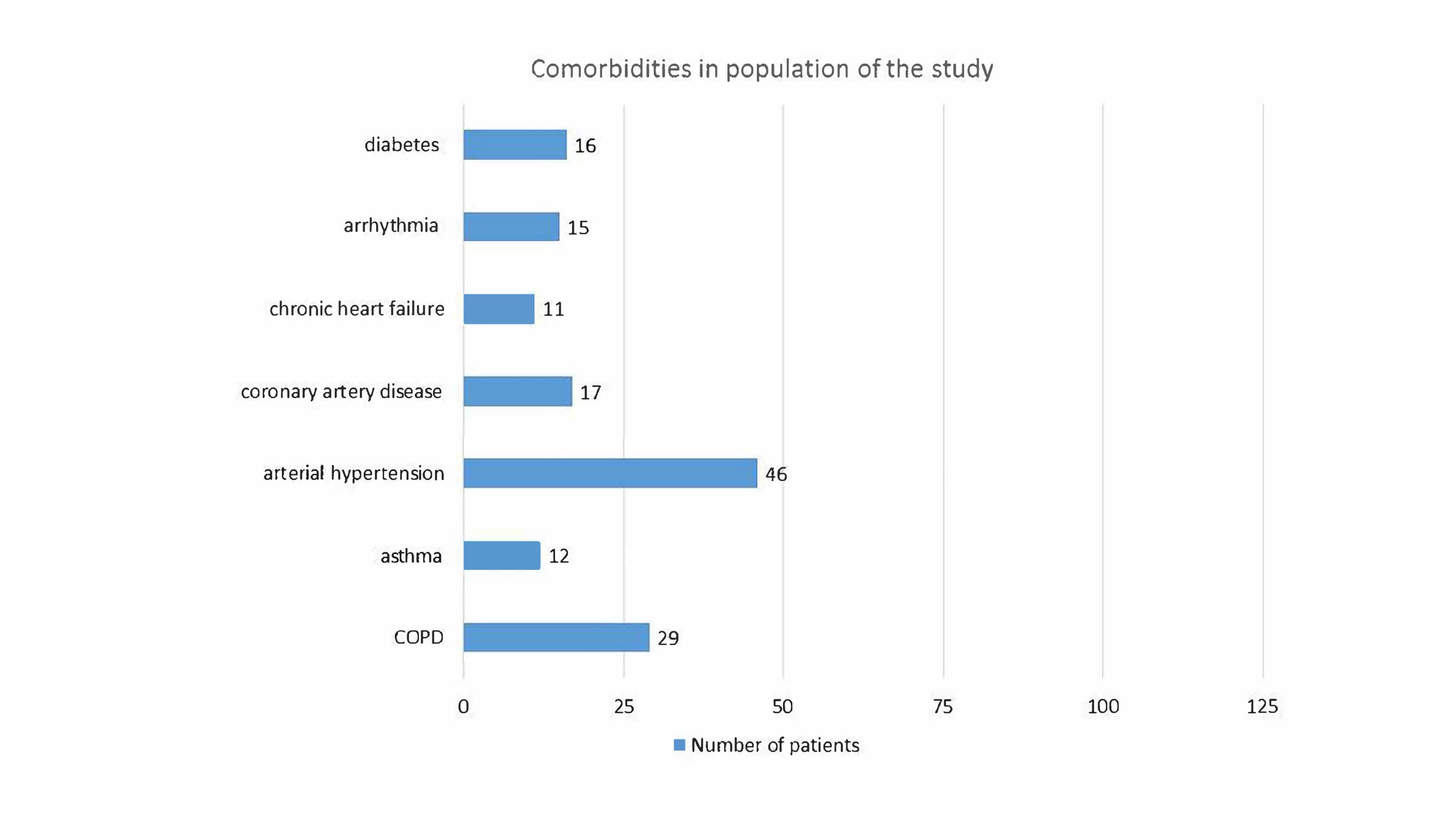

Supplement: S2 Fig — (TIF) [file pone.0274377.s006.tif]
