# Supplementary material for: Evaluation of patients’ satisfaction with bronchoscopy procedure
Source: PLoS One. 2022 Oct 6;17(10):e0274377. doi: 10.1371/journal.pone.0274377 (PMC9536568; doi:10.1371/journal.pone.0274377)
Supplement: Attachment — Submitted filename: Response to Reviewers.docx [file pone.0274377.s008.docx]

Warsaw, 10^th^ October 2021

Dear Editor and Reviewers,

Thank you for reviewing our manuscript PONE-D-21-03696, titled *Evaluation of patients’ satisfaction with bronchoscopy procedur*e. It is a great pleasure to learn that You found our manuscript potentially suitable for publication. We appreciate Your comments which have certainly helped us improve the manuscript’s quality. The revised version of the manuscript re-submitted for reevaluation includes changes made strictly according to Your suggestions. According to the Reviewers’ suggestion, we have excluded one group of patients and recalculated the results. We believe, that all the points raised by the reviewers were addressed. We would be grateful for re-considering our manuscript for publication.

Below, we include the specific responses to the Reviewers’ comments with the hope that they will find them adequate.

All references to the lines in the revised manuscript are referred to the file Revised Manuscript.TrackChages.

**Reviewer 1**

**Major points:**

*Reviewer’s comment*

1. Authors aimed to identify factors influencing patients’ satisfaction with BS. However, they did sample size calculations for correlation analysis to show that a sample size of 85 patients would provide 80% statistical power to detect weak (r=0.3) correlation (alpha = 0.05, beta = 0.20). In general, sample size calculation should be used to clarify the main objective. I wonder why they use correlation analysis to detect weak (r=0.3) correlation. Furthermore, did they do this sample size calculation in order to find out which factors correlated with which factors?

*Authors’ response*

Thank You for raising this issue. According to Your suggestion, we established a relationship (correlation) between patients’ anxiety and satisfaction with bronchoscopy as the primary outcome of the study. From our observations and based on previous studies we have found anxiety before procedure to be the most likely factor affecting patients’ satisfaction. Based on a study by Mitsumune et al. (Respirology 2005) we assumed that the correlation might be weak. If the correlation occurred stronger, the sample size would be lower. We based our calculation on a study by Bujang MA and Baharum N and added this article to references (revised manuscript, reference 13, line 646 ).

*Reviewer’s comment*

2. Among the results of this manuscript, I think the result of multivariate regression analysis which described in line 246 (revised manuscript line 374) is most important information. Authors concluded this result in the Conclusions session in line 379 (revised manuscript line 578). However, they included “amnesia due to premedication” in Discussion session in line 276 to 278 (revised manuscript line 423-426). If they included “amnesia due to premedication” from the results of Table 3, I think that “amnesia due to premedication” should be deleted because Table 3 included lots of confounds.

*Authors’ response:*

Thank You for this remark. According to the Reviewers’ suggestion, we excluded patients, who had bronchoscopy under general anaesthesia, which resulted in the change of some results (including those related to amnesia). Thus, amnesia due to premedication occurred to be a non-significant factor and has been deleted from both Results and Conclusions.

*Reviewer’s comment*

3. I think the result of multivariate regression analysis which described in line 246 (revised manuscript line 367) should be presented by additional table.

*Authors’ response*

Thank You for this comment. A new Table 3 was added according to the Reviewer’s suggestion.

*Reviewer’s comment*

4. In Statistical analysis and sample size calculation, the authors should present more detail on methods of multivariate regression analysis. That is, how they excluded the dependent factors from the selected factors and how they chose the best model among candidate models as final multivariate model.

*Authors’ response*

Thank You for this important remark. We added more information about regression analysis both in Statistical analysis and in Table 3.

*Reviewer’s comment*

5. In this study, questionnaire S1and S2 are very important. Questionnaire S1 include only two VAS on anxiety Q7, Q8. Questionnaire S2 include only one VAS on anxiety Q1. Authors used VAS for only anxiety scale. Other items were type of multiple-choice question. In case of multiple-choice question or Likert type question, they should validate them in order to detect the appropriate answers. Therefore, authors should give us the results of validation on these items.

*Authors’ response*

Thank You for this apt remark. Before starting the study, the questionnaires have been checked to whether they were comprehensible. However, we must admit that they were not fully validated. Therefore, we add this point to the study Limitations The Visual Analogue Scoring system has been applied according to the BTS guidelines and used for scoring patients’ anxiety and satisfaction.

**Minor points:**

*Reviewer’s comment*

1. Line 74 (revised manuscript line 102); “it is safe and severe complications are rare [1],”→Reference No.1 is too old. Please replace recent reference and recent information.

*Authors’ response*

Thank You for this comment. The ref#1 was replaced with the study by *Facciolongo N, Patelli M, Gasparini S, et al. Incidence of complications in bronchoscopy. Multicentre prospective study of 20,986 bronchoscopies. Monaldi Arch Chest Dis 2009* (revised manuscript, page 36, line 599).

*Reviewer’s comment*

2. Line 78 (revised manuscript line 108); “bronchoscopy is most commonly related to related to cough, dyspnea, chest pain or nausea” Is under lined part misprinted or not? Please correct the under lined part.

*Authors’ response*

Thank You for this comment, the duplicated phrase was delated (revised manuscript, page 6, line 108).

*Reviewer’s comment*

3. Line 99 (revised manuscript line 137); Does “elective bronchoscopy” mean “only diagnostic bronchoscopy”? If this is correct it should be defined in the text at first use.

*Authors’ response*

Thank You for this question. The term “elective bronchoscopy” has been used for non-emergency bronchoscopy procedures (diagnostic and therapeutic) which were scheduled in advance. This approach was necessary to have enough time to fulfil the questionnaire before bronchoscopy. In fact, the majority of them were diagnostic procedures, but therapeutic procedures such as endobronchial secretion removal or foreign body removal were also included (the latter have not been included in the current version of manuscript as it was performed under general anesthesia). In response to the Reviewer’s comment definition of the elective bronchoscopy was added to the manuscript (revised manuscript, page 7, line 137)

*Reviewer’s comment*

4. Line 108 (revised manuscript line 152); Authors mentioned “The inclusion to the study was not limited by the type of the procedure”. Considering statistical view, I think they should balance the type of the procedure when they enrolled patients.

*Authors’ response*

We agree with this comment. In response to this and one previous comment (#2) we excluded patients who underwent bronchoscopy under general anesthesia, thus excluding all rigid bronchoscopies. In consequence, the study group became significantly less heterogeneous in terms of the type of bronchoscopy procedures. The disparity between the number of videobronchoscopies and EBUS-TBNA procedures reflects the real proportion of these procedures in our department.

*Reviewer’s comment*

5. Line 129 (revised manuscript line 184); Outcome points had two points. Which is primary endpoint? Which is secondary endpoint?

*Authors’ response*

Thank You for this important remark. In reference to Your previous comments, we have modified the primary outcome. In the revised version, the primary outcome was to analyze the correlation between patients’ satisfaction with bronchoscopy and anxiety before it, while the secondary outcome was to identify other factors affecting patients’ satisfaction with the procedure (revised manuscript, page 10, line 184-188).

*Reviewer’s comment*

6. Line 136 (revised manuscript line 195); “Importantly, the staff had not been informed which patients were included to the study.” Why had the staff not been informed? Please explain this is important.

*Authors’ response*

Thank You for this comment. We did not inform the attending doctor, which patients agreed to take part in the study, as it might have influenced on the way of informing the patient about the procedure by the attending physician. It could have potentially led to bias. The wording was modified in the revised version of the manuscript to make it more clear (revised manuscript, page 10, line 195-197).

*Reviewer’s comment*

7. Line 157 and 159 (revised manuscript line 229 and 233); I think Table S3 and S4 are the clinical report form for bronchoscopists and attending physicians not questionnaire.

*Authors’ response*

Thank You for this point. We changed the titles of the Tables S3 and S4 (revised manuscript, page 12, line 227-233) according to this suggestion.

*Reviewer’s comment*

8. Line 182 to 193 (revised manuscript line 262 to 274); Authors mentioned background characteristics in this part. Please provide the table of “background characteristics” in text or Supporting Information.

*Authors’ response*

Thank You for this remark. We condensed the descriptive characteristics of the studied population in the manuscript text and added Figure 1 presenting indications for bronchoscopy and Figure 2 with comorbidities in study population in Supporting Information.

*Reviewer’s comment*

9. Line 200 to 204 (revised manuscript line 299-305); “Rigid bronchoscopy was performed in 6 patients (4%)”. Therefore, it would better that authors should compare only between flexible (fiberoptic) bronchoscopy without EBUS and that without EBUS.

*Authors’ response*

Thank you for this comment. We absolutely agree. As stated above, we excluded patients, who underwent rigid bronchoscopy under general anesthesia and recalculated the results.

*Reviewer’s comment*

10. In Table 1 “Satisfaction with BS in VAS”; Please consider the significant digits. “0 – 4.375” means they measured the degree of tens μm.

*Authors’ response*

Thank You for this apt comment. We changed the data in Table 1 according to Your suggestion.

*Reviewer’s comment*

11. Line 215 to 217 and 337 (revised manuscript line 318 to 321 and 511); How many patients achieved the conscious sedation? Depth of anesthesia is very important to evaluation the satisfaction with BS. Please provide depth of sedation by use of sedation score.

*Authors’ response*

Thank You for this valuable point. Unfortunately, we did not use sedation score during the study, so we are unable to analyze this factor. We can only explain that the sedatives were titrated to keep verbal contact with the patient.

*Reviewer’s comment*

12. Line 221 (revised manuscript line 327); Authors defined VAS > 6/10 as “very unsatisfied”. However, in Table 3 and Figure 2, they defined VAS ≥ 5-10 as “unsatisfied”. I think there is discrepancy. Please explained it. Furthermore, how did they define these cutoff-value?

*Authors’ response*

Thank You for this important remark. Indeed, this was our inaccuracy. We have corrected this by defining unsatisfied patients as those with VAS >5. We changed it in the text (revised manuscript, page 17, line 324) and Table 3 (revised manuscript, page 22). We ceased defining the “very unsatisfied” group. These cut-offs were set arbitrary to define two representative groups: the most satisfied and dissatisfied patients.

*Reviewer’s comment*

13. Line 223 and 238 to 240 (revised manuscript line 327 and 355 to 358); Was “correlation between satisfaction and willingness for future bronchoscopy” basis for setting for sample size calculations? Or was “correlation between dissatisfaction with bronchoscopy and the level of anxiety before the procedure” basis for setting for sample size calculations? Or was “correlation between dissatisfaction with bronchoscopy and patients discomfort during the procedure” basis for setting for sample size calculations? Which of them were basis for setting for sample size calculations? Was it primary endpoint?

*Authors’ response*

Thank you for this comment. Having regard to this and your previous remarks concerning sample size calculations and primary outcome, we clearly stated that the primary study outcome was the relationship between anxiety before bronchoscopy and satisfaction after the procedure. The assessment of other factors affecting the post-bronchoscopy satisfaction was pointed as the secondary outcome (revised manuscript, page 10, line 184-188).

*Reviewer’s comment*

14. Line 371 (revised manuscript line 564); “Secondly, the study group was rather small due to single-center study, inclusion criteria and short period of study, however the number of included patient was based on sample size calculation.” If authors set the main objective as sample size calculation, sample size of the study group would seem to be more appropriate size. Why did they use correlation analysis to detect weak (r=0.3) correlation?

*Authors’ response*

Thank You for this comment and question. We used correlation analysis to find whether there was any relationship between patient satisfaction with bronchoscopy and other factors. We assumed such a correlation might be weak based on the previous study by Mitsumune et al. (Respirology 2005), where correlation coefficient was R=0.376.

**Reviewer 2**

*Reviewer’s comment*

First of all, there are many grammatical errors in the text that needs to be corrected.

*Authors’ response*

Thank you for this comment. We performed double-checking of the manuscript and made every effort to eliminate all grammatical errors we could identify.

*Reviewer’s comment*

Secondly, the authors in justifying the use of oral route for bronchoscopy, made a statement of the nasal route being causing more discomfort. This justification is not accurate and the authors should just limit their statement to oral route being their preference.

*Authors’ response*

Thank you for this comment. We absolutely agree. Thus our own standpoint on the nasal route for bronchoscopy was deleted (revised manuscript, page 10, line 200-207)
