# Supplementary material for: Evaluation of patients’ satisfaction with bronchoscopy procedure
Source: PLoS One. 2022 Oct 6;17(10):e0274377. doi: 10.1371/journal.pone.0274377 (PMC9536568; doi:10.1371/journal.pone.0274377)
Supplement: Attachment — Submitted filename: Reviced paper_Commets and decision for author 20211106.docx [file pone.0274377.s009.docx]

Major revision _ Need additional analysis for further decision

There are still some issues as below.

1, Primary outcome: The authors set “a relationship (correlation) between patients’ anxiety and satisfaction with bronchoscopy as the primary outcome. They based their sample size calculation on a study by Bujang MA and Baharum N and provided this article to references. In this provided reference, Bujang MA and Baharum N used Pearson coefficient for correlation analysis. Did they check null hypothesis is equal to zero in this study population? Even if null hypothesis is equal to zero, I wonder why they use Mann Whitney U test or Kruskal- Wallis test that are non-parametric test. Why did they delete Kruskal- Wallis test in Statistical analysis of revised manuscript?

2, Primary outcome: I guess the authors described the result of primary outcome in Line 274 of Manuscript - revised manuscript, clean version.

The authors should describe this study met the primary outcome in Results or Discussion part.

3, Author should describe how to perform the multivariate analysis in this study more detail in in Statistical analysis part. what parameters were they screening by univariate analyses, selection criteria of parameters, how to find independent parameters, how to select best model. Please describe this process in Statistical analysis part.

4. In revised manuscript, you mention only regression model in univariate and multivariate. There are lots of regression method. I think you should not delete “logistic”.

5. In general, univariate analyses are only the screening test for model construction. Table 3 should include multivariate model like below table. However, you should check statistical and clinical independency between “Anxiety before BS” and “Discomfort during what”.

| Parameters | Univariate Analyses | | | Multivariate Analysis | | |
| --- | --- | --- | --- | --- | --- | --- |
|  | Beta | Standard error | *P-value* | Beta | Standard error | *P-value* |
| Anxiety before BS | 0.306 | 0.086 | 0.0006 | 0.264 | 0.086, | 0.003 |
| **Discomfort during ?** | 0.255 | 0.087 | 0.004 | 0.366, | 0.087 | 0.00017 |
| Age of patient | 0.015 | 0.090 | 0.864 | NI | | |
| Duration of BF | 0.039 | 0.092 | 0.674 | NI | | |
| Satisfaction with  information about the BF | -0.096 | 0.090 | 0.288 | NI | | |
| Not remembering BF | -0.136 | 0.090 | 0.135 | NI | | |

Abbreviations: NI, not included in the best multivariate logistic regression model.

6. Table 4 is “Differences between satisfied and unsatisfied patients” Why did not authors check all parameters in table 4 by univariate analyses? I think authors should be screening all parameters in table 4. And then they construct some models for the multivariate models. After that, they choose best model and analyze this model as the result. How is it?

7. Although author added the limitation, it is serious issue that they did not validate questionnaires used in the study.

8. There are lots of reports that depth of anesthesia is very important to evaluation the satisfaction with BS. It is serious issue that they did not use sedation score during the study. At least, they should add this point to the study Limitations.
