# Supplementary material for: Evaluation of patients’ satisfaction with bronchoscopy procedure
Source: PLoS One. 2022 Oct 6;17(10):e0274377. doi: 10.1371/journal.pone.0274377 (PMC9536568; doi:10.1371/journal.pone.0274377)
Supplement: Attachment — Submitted filename: Response to Reviewers - second review.docx [file pone.0274377.s010.docx]

Warsaw, 22^th^ December 2021

Dear Editor and Reviewers,

Thank you for reviewing our manuscript PONE-D-21-03696, titled *Evaluation of patients’ satisfaction with bronchoscopy procedur*e. We appreciate Your comments which have certainly helped us improve the manuscript’s quality. The revised version of the manuscript re-submitted for reevaluation includes changes made strictly according to Your suggestions. We believe, all the points raised by the reviews were addressed. We would be grateful for re-considering our manuscript for publication.

Below, we include the specific responses to the Reviewers’ comments with the hope that they will find them adequate.

- Primary outcome: The authors set “a relationship (correlation) between patients’ anxiety and satisfaction with bronchoscopy as the primary outcome. They based their sample size calculation on a study by Bujang MA and Baharum N and provided this article to references. In this provided reference, Bujang MA and Baharum N used Pearson coefficient for correlation analysis. Did they check null hypothesis is equal to zero in this study population? Even if null hypothesis is equal to zero, I wonder why they use Mann Whitney U test or Kruskal- Wallis test that are non-parametric test.

Thank you for this comment and suggestion. The manuscript by Bujang MA and Baharum N is a sample size guideline for Pearson correlation coefficient. However, when estimating for sample size, we could not predict whether the assumption for Pearson correlation would be met or not. Thus, our sample size analysis was based on estimation by Bujang et al.

In our study neither the satisfaction from bronchoscopy (BS) nor anxiety before BS have a normal distribution of values (line 203). Thus we changed the correlation coefficient for Spearman (line 209 and 287, 288) and we used non- parametric tests.

- Why did they delete Kruskal- Wallis test in Statistical analysis of revised manuscript?

Thank you for this remark. As we had excluded patients who had BS under general anesthesia, we compared only two groups (satisfied vs unsatisfied; midazolam vs midazolam +fentanyl; VBS vs EBUS) using Mann-Whitney U test ( Table 1,2,4).

- Primary outcome: I guess the authors described the result of primary outcome in Line 274 of Manuscript - revised manuscript, clean version. The authors should describe this study met the primary outcome in Results or Discussion part.

Thank you for this valuable comment. We changed the text (line 285) to emphasized the primary outcome of the study.

- Author should describe how to perform the multivariate analysis in this study more detail in in Statistical analysis part. what parameters were they screening by univariate analyses, selection criteria of parameters, how to find independent parameters, how to select best model. Please describe this process in Statistical analysis part.

Thank you for this comment. We screened all parameters from the table 4 (that were expressed in interval scale) in univariate analysis. Then we build multivariate linear regression model with backward stepwise analysis with all parameters and choose the model with optimal adjusted R square value (line 215-217).

- In revised manuscript, you mention only regression model in univariate and multivariate. There are lots of regression method. I think you should not delete “logistic”.

Thank you for this issue. We corrected the description of statistical methods for multivariate linear regression analysis (line 215-217).

- In general, univariate analyses are only the screening test for model construction. Table 3 should include multivariate model like below table.

| Parameters | Univariate Analyses | | | Multivariate Analysis | | |
| --- | --- | --- | --- | --- | --- | --- |
|  | Beta | Standard error | *P-value* | Beta | Standard error | *P-value* |
| Anxiety before BS | 0.306 | 0.086 | 0.0006 | 0.264 | 0.086 | 0.003 |
| Discomfort during procedure | 0.255 | 0.087 | 0.004 | 0.205 | 0.086 | 0.018 |
| Age of patient | 0.015 | 0.090 | 0.864 | NI | | |
| Duration of BF | 0.039 | 0.092 | 0.674 | NI | | |
| Satisfaction with  information about the BF | -0.096 | 0.090 | 0.288 | NI | | |
| Not remembering BF | -0.136 | 0.090 | 0.135 | NI | | |

Abbreviations: NI, not included in the best multivariate logistic regression model.

Thank you for your comment and suggestion, which is very helpful. We changed Table 3 according to your proposal.

- However, you should check statistical and clinical independency between “Anxiety before BS” and “Discomfort during procedure”.

We found very low non-significant correlation between anxiety prior to BS and discomfort during the procedure (r=0.173, p=0.055), so we used both factors in model of multivariate regression analysis.

- Table 4 is “Differences between satisfied and unsatisfied patients” Why did not authors check all parameters in table 4 by univariate analyses? I think authors should be screening all parameters in table 4. And then they construct some models for the multivariate models. After that, they choose best model and analyze this model as the result. How is it?

Thank you for your suggestion. We screened all parameters with univariate analysis finding only two significant parameters (anxiety before BS and discomfort during procedure), which were selected for multivariate logistic regression analysis.

- Although author added the limitation, it is serious issue that they did not validate questionnaires used in the study.

Thank you for your comment. Indeed, it is an important issue. We emphasized it in limitation of the study (line 489)

- . There are lots of reports that depth of anesthesia is very important to evaluation the satisfaction with BS. It is serious issue that they did not use sedation score during the study. At least, they should add this point to the study Limitations.

Thank you for your important remark. We added it to limitations of the study (line 490-491)
