# Supplementary material for: Evaluation of patients’ satisfaction with bronchoscopy procedure
Source: PLoS One. 2022 Oct 6;17(10):e0274377. doi: 10.1371/journal.pone.0274377 (PMC9536568; doi:10.1371/journal.pone.0274377)
Supplement: Attachment — Submitted filename: Response to Reviewers.February 2022.docx [file pone.0274377.s011.docx]

Warsaw, 15^th^ February 2022

Dear Editor and Reviewers,

Thank you for reviewing our manuscript PONE-D-21-03696, titled *Evaluation of patients’ satisfaction with bronchoscopy procedur*e once again. We truly appreciate Your comments which have helped us improve the manuscript’s quality. We believe that all the points raised by the reviewer were addressed. We would be grateful for re-considering our manuscript for publication.

Below, we include the specific responses to the Reviewers’ comments with the hope that they will find them adequate.

1) “In our study neither the satisfaction from bronchoscopy (BS) nor anxiety before BS have a normal distribution of values (line 203). Thus we changed the correlation coefficient for Spearman (line 209 and 287, 288) and we used non-parametric tests.”
Does this mean sample size calculation was incorrect in your study? You should include this information as a limitation. This is very important.

Thank you for this remark. We added this information in limitations of the study (line 201-223).

2) “The primary outcome of the study was a week positive correlation between dissatisfaction with bronchoscopy and the level of anxiety before the procedure (Spearman coefficient r=0.276, p=0.0014) or patients’ discomfort during the procedure (r=0.309, p=0.0005)” Although the correlation between dissatisfaction with bronchoscopy and the level of anxiety before the procedure was statistically significant, coefficient rho was 0.276 that was below 0.3. On the other hand, the correlation between dissatisfaction with bronchoscopy and patients’ discomfort during the procedure was statistically significant and its coefficient rho was 0.309 that was beyond 0.3. Is the latter the only one that met the main outcome? You should explain this result in the discussion part.

Thank you for your suggestion. We emphasized that correlations were weak, but according to your suggestion we drew more attention to the correlation between dissatisfaction and discomfort (line 406-407). We tried to discuss the significance of discomfort as a factor influencing patients satisfaction with bronchoscopy in detail (line 400-421).

3) I think the coefficient is generally rho (ρ) in the Spearman rank test.

Thank you for this remark. We corrected it for rho (line 286-287).

4) Although PLOS one is an open-access journal, the discussion part is slightly long. Can you summarize it?

Thank you for this hint. We tried to shorten the discussion.

5) I have a comment for your future study. There is a book that explained how to use Multivariable Analysis: A Practical Guide for Clinicians and Public Health Researchers Second edition by Mitchell H. Katz. He said as below: Whenever possible, "do not use variable selection technique." This is because there is a danger that any variable selection method will select confounders into the model and remove variables that are causally related to the outcome. Also, in both the forward and the backward selection, each variable is evaluated individually, so there is a possibility that two variables that start out as a set and have an important effect on the outcome will not be selected as a set in the final model. There is also the possibility that a variable that is very important in explaining the outcome may not be selected for the model because it is related to a variable that has already been adopted in the model. My opinion is the same as above.

Thank you for this remark. We appreciate all comments and suggestion concerning the statistical analysis. We will certainly read this book and use this knowledge in future studies.

6) This manuscript still has some grammatical errors in the text that needs to be corrected. Please check English in the text carefully.

Thank you for this suggestion. We checked and corrected the manuscript.

Yours sincerely

Katarzyna Faber

Marta Dąbrowska
