# Supplementary material for: Evaluation of patients’ satisfaction with bronchoscopy procedure
Source: PLoS One. 2022 Oct 6;17(10):e0274377. doi: 10.1371/journal.pone.0274377 (PMC9536568; doi:10.1371/journal.pone.0274377)
Supplement: Attachment — Submitted filename: Response to Reviewers.March 2022.docx [file pone.0274377.s012.docx]

Warsaw, 14^th^ March 2022

Dear Editor and Reviewers,

Thank you for reviewing our manuscript PONE-D-21-03696, titled *Evaluation of patients’ satisfaction with bronchoscopy procedur*e once again. We are grateful for Your comments and suggestions which have helped us improve the manuscript’s quality. We hope that all the points raised by the reviewer were addressed. We would be grateful for re-considering our manuscript for publication.

Below, we included the specific responses to the Reviewers’ comments with the hope that they will find them adequate.

#1; In Manuscript – revised version. February 2022.
Line 260 to 262; “There was a positive correlation between satisfaction and willingness for future bronchoscopy (r=0.487, p<0.0001)”.
I wonder if this “r” is Spearman coefficient rho or not. Please correct or explain it.

Response:

Thank you for this apt remark. In line 260-262 Pearson correlation r=0.487, p< 0.0001 was given, but as distribution of data were not normal, we changed it for Spearman correlation rho= 0.404, p<0.0001 (p=0.000003).

#2; And you also need clear and correct Figures and Tables without any misprints (“diabetes; diabetes mellitus,” “arhytmia; arrhythmia,” “asthma; bronchial asthma” in Supplementary Figure 2 and so on) or any additional linear (Q1 in S2 table and so on) to publish. Therefore, you should check all figures and tables and supplementary tables and figures again.
Response:

Thank you for a thorough review. We have revised the supplementary files and tabels and provided the required changes.

#3; According to Submission Guidelines, manuscripts must be submitted in English. Your supporting information included the Polish version of the S1 Table to S4 Table. Therefore, you should delete them.

Respone:

Thank you for this remark. Files in Polish have been deleted.

Thank you for those suggestions.

Yours sincerely

Katarzyna Faber

Marta Dąbrowska
